# ‘Early identification of struggling pre-clerkship learners using formative clinical skills OSCEs: an assessment for learning program.’

**DOI:** 10.1080/10872981.2022.2028333

**Published:** 2022-01-20

**Authors:** Ilene Rosenberg, Listy Thomas, Gabbriel Ceccolini, Richard Feinn

**Affiliations:** a Clinical Skills Remediation, Department of Medical Sciences, Frank H. Netter Md School of Medicine at Quinnipiac University, Hamden, CT, USA; b Department of Medical Sciences, Clinical Arts and Sciences Course, the Frank H. Netter Md School of Medicine at Quinnipiac University, Hamden, CT, USA; c Standardized Patient & Assessment Center, Frank H. Netter Md School of Medicine, at Quinnipiac University, Hamden, CT, USA; d Department of Medical Sciences, Frank H Netter Md School of Medicine, at Quinnipiac University, Hamden, CT, USA

**Keywords:** Early identification, formative OSCE, remediation of clinical skills, clinical skills assessment

## Abstract

Multiple experts in clinical skills remediation recommend early identification to support struggling learners, but there is minimal documentation on implementation of these programs. We share one school’s outcomes-based research utilizing the formative assessment for learning model to early-identify pre-clerkship students struggling with clinical skills using formative OSCEs (F-OSCE). Student scores were monitored over longitudinal F-OSCE experiences as part of a curricular innovation. Points towards early identification accumulated when a student’s score fell below the 80% threshold for each section of an OSCE. Students who accumulated enough points were advised of the need for intervention, and coaching was recommended. Students were surveyed about their experiences with the program. The objective was to explore whether this early identification program and coaching intervention had a positive impact on subsequent OSCE performance. Of 184 students in 2 cohorts who completed F-OSCEs, 38 (20.7%) were flagged for early identification. Of these, 17 (44.7%) sought additional help by voluntarily participating in the coaching program. Students who participated in extra clinical skills coaching demonstrated statistically significant improvements in performance on subsequent FOSCEs, as did the early identified students who did not participate in extra coaching. The greatest impact of coaching intervention was noted in the physical examination domain. This program was effective in identifying students struggling with clinical skills on formative OSCEs. Early identified students demonstrated improvements in subsequent OSCE performance, with those who sought coaching faring slightly better. Development of robust early identification programs as formative assessments of clinical skills and follow-up coaching programs to guide skills development are important implications of this work. Monitoring short- and long-term results for students identified through this approach to see if improvement is sustained is planned.

## Introduction

‘An ounce of prevention is worth a pound of cure.’ – Benjamin Franklin.

The traditional approach to assist struggling learners – remediation – often is more reactive than proactive. Assistance is offered to students who have already failed, rather than identifying students who are struggling and providing the needed learning support early to prevent future failure. Additionally, students who are struggling and most in need of support often fail to seek help [1] before failure. Low academic performance in the first year of medical school is a meaningful risk factor with both predictive validity and utility for low performance later in medical school, encompassing both knowledge and clinical skills [2]. Up to 15% of third-year medical students struggle during their clerkships, up to 11% struggle during the fourth year and 8–15% of residents have significant areas of learner difficulty with little variation among specialties [3]. Struggling students report lower task value and self-efficacy, boredom, and higher frustration and anxiety [4]. Students identified later in medical education have a higher likelihood of presenting with a lack of confidence and discouragement and a lag in academic performance which could have a potentially negative impact on their ability to make meaningful changes in study habits and practices [5]. Position papers recommend that services for underperforming learners should be a regular part of medical education, versus an external activity [6–8], with one suggesting ‘zones of engagement’, encompassing the ‘normal’ curriculum, corrective action, remediation, probation, and exclusion [8].

Given the recent cancellation of the USMLE Step 2 Clinical Skills Exam, the necessity of increased rigor in clinical skills assessments such as formative and summative OSCE’s to demonstrate competency is clear. It also raises the imperative that schools must utilize all available resources to assist struggling learners in this new environment. There is growing consensus that early identification and intervention are important for improving students’ skills. Earlier identification and assistance to learners not meeting qualification standards has been discussed by many but is rarely implemented in a systematic manner[9]. While a variety of services exist across medical schools [10], many remediation programs focus on medical knowledge rather than clinical skills. It has been shown that formative assessment systems are most useful to students when the system is a clearly defined process that connects performance events and relays information to the student [11]. Additionally, variability in supervision of bedside clinical experiences in the pre-clerkship phase of medical education may impede reliable early identification of students with clinical skill deficits [12,13]. Thus, we identified the formative OSCE (f-OSCE) as an optimal way to identify struggling students. The OSCE was developed by Harden et al. to improve the assessment of clinical competencies [14] and the purpose of this study was to evaluate the effectiveness of early identification of struggling students through a point system and coaching intervention.

## Methods

The study took place in the Clinical Arts and Sciences course at the Frank H. Netter MD School of Medicine at Quinnipiac University. Medical students in their first and second year experience a series of formative OSCEs, followed by a summative-OSCE at the end of each academic year. First and second year medical students participate in six required multi-station formative OSCEs that integrate with the academic content being taught in each basic science block, followed by an end-of-year 5-station summative-OSCE. Stations include 20 minutes with the Standardized Patient (SP) followed by 5-minutes of dedicated SP verbal feedback before the student rotates to the next station. This allows for a formal formative feedback system in which all undergraduate medical education students participate to enhance student performance on future assessments. SP’s score the students on history taking, physical exam, and communication using predefined checklists.

### Development of OSCE checklists

In consultation with other standardized patient programs and faculty experts, 30-item checklists were created for each OSCE in our clinical skills course. This was determined to be a reasonable number of items an SP could remember and complete in a 5-minute window [15]. Items were split in the categories of history, physical and communication and included a mix of case-specific and generic items. Case-specific checklists provided detailed information about learners’ skills with medical history taking and physical examination [16]. Items for history and physical focused on assessing thoroughness and task completion and aligned with class instruction. Faculty expert consensus determined items to include in the checklist for particular cases, such as smoking for cough or alcohol use for abdominal pain. Communication items consisted of a standard set of 10–12 items from the Master Interview Rating Scale (MIRS) [17] which assesses general communication skills. Checklists are reviewed yearly and adjusted with standard setting exercises, utilizing faculty and SP feedback and descriptive measures such as case-item analysis. Students have access to their performance on these checklists and their OSCE videos to review after each formative OSCE encounter.

### Early identification program

While OSCES are used extensively in medical school, methods for setting minimum competency standards vary widely and there is no consensus on the best approach to identify students for added learning interventions [18,19]. For this early identification program, minimum competency standards were defined using benchmarks of historical failure scores on the Summative OSCE taken at the end of the second year. Students must pass each section of the assessment: history (Hx), physical exam (PE) and communication (COM). A failure score is typically set at around 2(SD) below the mean, utilizing a Hofstee [20] method of compromised absolute and relative measures. The historical passing scores of the Y2 summative OSCE are shown in Table 1. The Y1 passing scores are similar.

**Table 1.** Y2 S-OSCE passing scores

	Class of 2018	Class of 2019	Class of 2020	Class of 2021
Physical exam	77%	78%	78%	85%
Communication	%	84%	84%	87%
History	87%	90%	88%	90%

When considering the appropriate cutoff scores for early identification, the Hofstee method was used to define a score of 80% as the minimum level of competency a student should achieve on each section of an OSCE, independent of cohorts. Analysis of data for the first cohort demonstrated the 80% cut-off was very close to 2(SD) below the mean for the history and communication section of the formative OSCEs. For the physical exam, scores 2(SD) below the mean were closer to 70%. This is similar to the cut-off scores found on summative OSCEs.

Points accumulated when a student’s score fell below the 80% threshold for each section of an OSCE: history, physical exam, and communication. Two points were allocated each time a student scored below the cutoff of 80% in the history section and 80% in the communication section. Given the lower cut-off score for physical exam component, students accumulate one point at the 80% cutoff and 2 points when the score was 70% or below. Scores significantly below these performance levels were assigned additional points. Point allocations are shown in Table 2.

**Table 2.** Point allocations for low performance on the FORMATIVE OSCE

History	Physical exam	Communication
Score <80 = 2 points Score <60 = 4 points	Score <80 = 1 point Score <70 = 2 points Score <60 = 3 points	Score <80 = 2 points Score <60 = 4 points

### Data collection and student identification

Data was collected after each formative OSCE between 2017 and 2019. In order to identify struggling students early based on formative OSCE assessments that had no standardized cut-offs in place, we devised a point system that allowed for longitudinal monitoring of the students’ progress across multiple OSCEs. Once a student accumulated 6 points, in any of the domains of history, physical exam, or communication skills, the student was identified as

possibly needing additional coaching in clinical skills. The 6-point threshold for identification was chosen to account for test variability and to ensure that the problem was reproducible and not specific to a given OSCE in time. Multiple assessments would be needed to reach the 6-point threshold unless a student struggled in multiple domains. Students who accumulated 6 points received written notification from the course of their trailing formative OSCE performance and offering additional practice and coaching to further their clinical skills development. Because the assessments were formative, and in order to remain learner-centered and utilize the assessment for learning framework [11], students were not required to participate in the coaching. Instead, clinical skills coaching was strongly recommended by the course leadership and students were allowed to utilize this extra resource at their own discretion.

### The coaching process with the clinical skills coach

Students who were identified could voluntarily meet with the clinical skills coach.

This was done by the clinical skills coach on an individual basis. Several processes utilized were video review with direct feedback and reflection, deliberate practice of communication, history and PE practice with either a fellow student and/or SP with direct observation and feedback from the clinical skills coach. The domain(s) that the student collected points in were the areas of concentration during these sessions.

The number of sessions varied from 1 to 10 sessions per student with an average of about 2 sessions per student.

The process in which their preceptors or other people helping the students in clinical skills was not monitored.

### Student survey

Student feedback on program effectiveness solicited using a Likert scale survey. The survey was sent at the completion of the academic year to all second-year students who were identified through the program. Participating was anonymous and voluntary. The survey is provided as Electronic Supplemental Material 1.

This study was approved by the Frank H Netter/Quinnipiac University Institutional Review Board.

## Analysis

We used linear mixed models to assess the effect of early identification on subsequent formative OSCE scores [21]. The outcome, students’ formative OSCE scores, was modeled by an indicator variable that was coded 0 for scores (blocks) prior to identification and 1 for scores after identification using our points system. The class average for that block’s formative OSCE was included as a covariate to adjust for the difficulty level for that block’s exam. Thus, the coefficient for the indicator variable denotes how much the formative OSCE score differed for students after they had been identified compared to before being identified, adjusting for the overall difficulty of the exam.

A second linear mixed model analysis was used to assess the effect of students participating in the clinical skills coaching that was being offered to those who had been identified. We coded 0 for students who decided not to participate in the formal clinical coaching program and 1 for students who did participate in this coaching. The interaction between identification and coaching tested if there was a difference in subsequent formative OSCE scores among students who engaged in the coaching program versus student who did not engage in the program.

To identify which domain of the formative OSCE (history, physical exam, and communication) was affected most by student identification and subsequent help-seeking, we used a multivariate linear mixed model that simultaneously modeled the three outcomes. The mixed models included fixed effects for the indicator variable and class average and a random intercept that varied by student. For the multivariate model, we added random terms for each of the three subscales and applied an unstructured covariance matrix to the random effects. Analyses used SPSS v25 and the alpha level for statistical significance was set at 0.05.

## Results

The Netter Early Identification program (Early ID) was created for first- and second year students. Data presented in this study specifically represent a second-year sample. All second-year medical students from the classes of 2020 and 2021 participated in the formative OSCEs. Thirty-eight students (20.7%) were identified and contacted due to performance below the cut-off score and 17 of those students (17/38, 44.7%) sought the additional help that was advised (Table 3). Because of the conservative longitudinal point accumulation, most students (14/38, 36.8%), were identified by December of the academic year since it took about three OSCEs for most students to accumulate 6 points. Of the 38 students identified, only two failed the end of year summative OSCE. One of these students had chosen to seek added help, the other had not. In most cases, the early identification program flagged recurrent longitudinal deficiencies, as suggested by earlier studies [22].

**Table 3.** Students identified and students who sought additional help

FOSCE	Students identified (individual F-OSCE)	Sought additional help (after being identified)
1	0	0
2	2	0
3	7	1
4	14	7
5	6	7
6	7	2
Total	38	17

We did not use data from the first year due to a limited sample size of identified students. In both cohorts of year-one data that we collected, first year students did not meet early identification thresholds until the last FOSCE before the Summative OSCEs. Therefore, none of those students had an opportunity to participate in consistent coaching before the SOSCE.

In Table 3 FOSCE 5, an additional student who had been identified previously sought help.

Table 4 (see Electronic Supplementary Material 2) shows the estimated means from the linear mixed models. There was a significant increase in subsequent F-OSCE scores after students were identified and notified of their performance (F1,247 = 6.52, p = .011) with an estimated increase in score of two points, after adjusting for class average scores. Students who participated in extra clinical skills coaching increased their scores by 2.5 points, and students who did not participate in the extra practice also increased their scores by 1.6 points, but this difference was not statistically significant (F_1,241_ = 0.50, p = .480).

To better understand the locus of the effect of the Early ID program we ran a multivariate linear mixed model.

Table 5 (see Electronic Supplementary Material 3) shows the coefficients from the model, which represent the change in score before and after being identified. While both communication and history scores increased, this change was not statistically significant. There was a significant change (t = 3.68, p < 0.001) in the physical exam subscale with scores increasing 4.5 points (5% increase) after identification. A multivariate mixed model testing for the effect of seeking additional help showed no significant effect for any of the three domains.

Student Survey

Eighteen second year medical students identified completed the survey (18/38, 47.4%). The majority of these students (n = 16, 89%) agreed implementing the Early ID program was a good decision. Fourteen students (78%) reported Early ID was beneficial; however, 10 (55%) reported it as stigmatizing. Of the students who did not seek coaching 5 (50%) felt that they could correct the problem on their own,2 (20%) practiced with a peer, 1(10%) felt too embarrassed to ask anyone for help and 2 (20%) responded as other. Of the 14 students who received extra clinical skills coaching, 70% worked with the clinical skills coach, 24% worked with their group preceptors and 1% worked with course leadership. Of these students 12 (86%) thought it was helpful. Fourteen students (78%) reported they changed their approach to OSCE’s as a result of the coaching and 3 (17%) reported they changed their approach to the school’s longitudinal clinical experience. Most (n = 16, 89%) thought clinical skills remediation after being identified should not be mandatory.

## Discussion

Early identification has been largely touted as critical in remediation processes [23], but there is limited literature on the specifics of what an early identification program using longitudinal formative assessment could look like. This is one of the first studies for pre-clerkship medical students that identified students with formative (not summative) exams and broke the issues into different domains (communication, history, physical exam). We wanted to create a method that was simple to utilize using a point system. The Netter School of Medicine Early ID program was created and implemented to enable targeted early identification of specific domains of clinical skills competency among medical students participating in longitudinal formative OSCEs to support an assessment for learning paradigm.

This program identified 21% of second year medical students with scores below school-established cut-off score on formative OSCE . Of this group, nearly 45% sought further assistance with skills development. Linear improvement in successive formative OSCE performance and successful passing of the summative OSCE were metrics used to validate this early identification program.

Interestingly, students showed improvement in subsequent OSCE performance, even if they did not seek additional clinical skills coaching. Several reasons could explain this. Struggling students often have inappropriate learning strategies, inflated beliefs about their performance and contribute external reasons for not doing well as a coping strategy[24]. Identification could be a wake-up call for students that they are not performing up to par. Insight into areas of potential improvement is often lacking among struggling medical students [25–27] and notification may offer additional sources of feedback that these students can build upon. This is consistent with prior literature demonstrating that identification of these lower scored performances on formative assessments may be enough to inform potentially struggling students of the need for self-assessment and self-improvement [28]. Just by being identified, students were able to self-improve based on qualitative data from surveys, and data from subsequent FOSCES.

Students who participated in coaching tended to fare better although this was not statistically significant. There is a lack of rigorous quantitative research into medical coaching programs. However, in a review by Lovell [29], strong evidence to support coaching as a method to improve technical skills was noted mostly in the domain of surgical skills. Further studies need to be done on the benefits of coaching programs for clinical skills.

Given the voluntary nature of participation in the early identification program, reasons clarifying why some students sought further coaching and some did not are not readily available [30]. After being identified, students could have practiced skills on their own or sought help from other faculty members or peers. The most significant area for improvement after identification was the physical exam component. The reasons are not clear. Because the scoring was lower for this competency among those early identified, there might have been more room for improvement. Data from our student surveys showed students who were identified via the Early ID program and participated in coaching had positive attitudes towards the program and the majority reported they changed their approach to the OSCE as a result of it.

We thought the Hofstee method was the best approach as it reflects how we determine performance, which is based on expert opinion on what qualifies as acceptable and performance among peers. This seemed most practical considering scores can vary by block making establishing predetermined cut-points across blocks difficulty.

To our knowledge, this is one of the first studies for pre-clerkship medical students that identified students with formative (not summative) exams and broke the issues into different domains (communication, history, physical exam) and therefore there is limited research for comparative analysis.

Our study has limitations. It was conducted at a single institution with a limited sample and results may not generalize to other settings. Because the decision to seek remediation after being identified is elective, it is not clear if there would be greater and/or more sustained improvement if participation was mandatory. We also need to change the culture in which students identified do not feel targeted and the need for improvement is not seen as negative. We believe that this is one of the reasons that a number of students did not seek coaching. Intentionality in the naming of the framing of the intervention as a coaching rather than remediation opportunity aids in the de-stigmatization of seeking additional help. We have also started to implement peer to peer clinical skills coaching sessions under the direction of the director of clinical skills coach to further help destigmatize this process. We do not have data yet whether this will have an impact. Interestingly, those who sought coaching felt that it was helpful. Whether there will be a lower cut-off in which participation in remediation is required needs to be determined. Our study was small and did not reach statistical significance for coaching except in the physical exam domain although the trends seemed to indicate improvement. Although the identification program itself might be enough to make students aware that there was an issue that they needed to work on, we believe that larger studies will demonstrate a statistically significant benefit of coaching especially as we work on creating an environment in which coaching is not stigmatizing.

Future plans for the early identification program study center around monitoring students longitudinally through their clerkships to determine if the appropriate cohort of students are captured in this program and if the improvements seen in the formative OSCEs are maintained. We also plan to explore broader models of learning support for underperforming students, including continued involvement, and coaching through the clinical phase of medical school. We plan to retrospectively survey the identified students who are now in their clerkships regarding their readiness for clerkship and whether the Early ID program helped them in this transition. Finally, we will explore how students changed their approach to clinical skills acquisition, and whether these changes were sustained. We hope to expand our early identification program to Clerkship OSCEs and further refine our process and interventions.

This early identification program can be implemented as a practical tool for clinical skills programs that utilize formative OSCEs. Early identification for clinical skills competencies without further interventions may have benefits for students’ awareness of their performance and the pursuit of self-regulated learning.

## Supplementary Material

Supplemental Material

## References

[1] Winston KA, Van Der Vleuten CP, Scherpbier AJ. At‐risk medical students: implications of students’ voice for the theory and practice of remediation. Med Educ. 2010;44:1038–7. 20880373 10.1111/j.1365-2923.2010.03759.x

[2] Krupat E, Pelletier SR, Dienstag JL. Academic performance on first-year medical schoolexams: how well does it predict later performance on knowledge-based and clinical assessments? Teach Learn Med. 2017;29(2):181–187. 28098483 10.1080/10401334.2016.1259109

[3] Guerrasio J. The need for remediation. In: Guerrasio J, editor. Remediation of the struggling medical learner. 2nd. Association of Hospital Medical Education; 2018. p. 7. 2nd edition. Chapter 1.The need for remediation

[4] Artino AR Jr, Hemmer PA, Durning SJ. Using self-regulated learning theory to understand the beliefs, emotions, and behaviors of struggling medical students. Acad Med. 2011;86(10):S35–8. 21955765 10.1097/ACM.0b013e31822a603d

[5] Chou CL, Kalet A, Hauer KE. A research agenda for remediation in medical education. In: Kalet A, Chou CL, editors. Remediation in medical education: a mid-course correction. NewYork: Springer Hill; 2014. p. 339–348. DOI:10.1007/s40037-017-0385-6

[6] Bennion L, Durning S, Larochelle J, et al. Untying the gordian knot: remediation problems in medical schools that need remediation. BMC Med Educ. 2018;18(1). DOI:10.1186/s12909-018-1219-x. PMC5984332 29855302

[7] Kalet A, Chou C, Ellaway R. To fail is human: remediating remediation in medical education. Perspect Med Educ. 2017;6. DOI:10.1007/s40037-017-0385-6 PMC5732108 29071550

[8] Ellaway RH, Chou CL, Kalet AL. Situating remediation: accommodating success and failure in medical education systems. Acad Med. 2018;93(3):391–398. 28767496 10.1097/ACM.0000000000001855

[9] Bennion LD, Durning SJ, LaRochelle J, *et al*. Untying the gordian knot: remediation problems in medical schools that need remediation. BMC Med Educ. 2018;18:120. 29855302 10.1186/s12909-018-1219-x PMC5984332

[10] Hinman PG, Dottl S, Passon J. Academic development: a survey of academic difficulties experienced by medical students and support services provided. Teach Learn Med. 2018;21(3):254–260. 10.1080/10401330903021041 20183347

[11] Konopasek L, Norcini J, Krupat E. Focusing on the formative: building an assessment system aimed at student growth and development. Acad Med. 2016;91(11):1492–1497. 27028028 10.1097/ACM.0000000000001171

[12] Kassebaum DG, Eaglen RH. Shortcomings in the evaluation of students’ clinical skills and behaviors in medical school. Acad Med. 1999;74:842–849. 10429595 10.1097/00001888-199907000-00020

[13] Howley LD, Wilson WG. Direct observation of students during clerkship rotations: a multiyear descriptive study. Acad Med. 2004;79:276–280. 14985204 10.1097/00001888-200403000-00017

[14] Harden RM, Stevenson M, Downie WW, et al. Assessment of clinical competence using objective structured examination. Br Med J. 1975;1(5955):447–451. 1115966 10.1136/bmj.1.5955.447 PMC1672423

[15] Hurley KF, Giffin NA, Stewart SA, et al. Probing the effect of OSCE checklist length of inter-observer reliability and observer accuracy. Med Educ Online. 2015;20:29242. 26490948 10.3402/meo.v20.29242 PMC4613902

[16] Gorter S, Rethans J, Scherpbier A, et al. Developing case-specific checklists for standardize-patient – based assessments in internal medicine: a review of the literature. Acad Med. 2000;75(11):1130–1137. 11078676 10.1097/00001888-200011000-00022

[17] Stillman PL, Brown DR, Redfield DL, et al. Construct validation of the Arizona clinical interview rating scale. Educ Psychol Meas. 1977;37(4):1031–1038.

[18] Boursicot K. Setting standards in a professional higher education course: defining the concept of the minimally competent student in performance‐based assessment at the level of graduation from medical school. Higher Educ Q. 2006;60:74–90.

[19] Boursicot K, Etheridge L, Setna Z, et al. Performance in assessment: consensus statement and recommendations from the Ottawa conference. Med Teach. 2011;33(5):370–383. 21517685 10.3109/0142159X.2011.565831

[20] Hofstee WKB. The case for compromise in educational selection and grading. In: Anderson SB, Helminck JS, editors. On educational testing. San Francisco: Jossey-Bass; 1983. p. 109–127.

[21] Fitzmaurice GM, Ravichandran C. A Primer in longitudinal data analysis. Circulation. 2008;118:2005–2010. 18981315 10.1161/CIRCULATIONAHA.107.714618

[22] Boscardin CK. Profiling students for remediation using latent class analysis. Adv in Health Sci Educ. 2012;17:55–63. 10.1007/s10459-011-9293-4 21487932

[23] Winston KA, Van Der Vleuten CPM, Scherpbier AJJA. Prediction and prevention of failure: an early intervention to assist at-risk medical students. Med Teach. 2014;36:25–31. 24083365 10.3109/0142159X.2013.836270

[24] Patel R, Tarrant C, Bonas S, et al. The struggling student: a thematic analysis from the self-regulated learning perspective. Med Educ. 2015;49:417–426. HYPERLINK “10.1111/medu.12651 25800302

[25] Martin IG, Jolly B. Predictive validity and estimated cut score of an objective structured clinical examination (OSCE) used as an assessment of clinical skills at the end of the first clinical year. Med Educ. 2002;36:418–425. 12028391 10.1046/j.1365-2923.2002.01207.x

[26] Klamen DL, Borgia PT. Can students’ scores on preclerkship clinical performance examinations predict that they will fail a senior clinical performance examination? Acad Med. 2011;86(4):516–520. 21346501 10.1097/ACM.0b013e31820de435

[27] Kruger J, Dunning D. Unskilled and unaware of it: how difficulties in recognizing one’s own incompetence lead to inflated self-assessments. J Pers Soc Psychol. 1999;77(6):1121–1134. 10626367 10.1037//0022-3514.77.6.1121

[28] Bernard AW, Ceccolini G, Feinn R, et al. Medical students review of formative OSCE scores, checklists, and videos improves with student-faculty debriefing meetings. Med Educ Online. 2017;22(1):1324718. 28521646 10.1080/10872981.2017.1324718 PMC5508650

[29] Lovell B. What do we know about coaching in medical education? A literature review. Med Educ. 2018;52:376–390. 29226349 10.1111/medu.13482

[30] Coelho C, Zahra D, Ali K, et al. To accept or decline academic remediation: what difference does it make? Med Teach. 2019;41(7):824–829. 30942639 10.1080/0142159X.2019.1585789

