# Supplementary material for: ‘Early identification of struggling pre-clerkship learners using formative clinical skills OSCEs: an assessment for learning program.’
Source: Med Educ Online. 2022 Jan 20;27(1):2028333. doi: 10.1080/10872981.2022.2028333 (PMC8786239; doi:10.1080/10872981.2022.2028333)
Supplement: Supplemental Material [file ZMEO_A_2028333_SM1695.zip › Supplementary files/Electronic Supplementary Material 1.docx]

**Survey for Students Early Identified**

**Glossary of Terms**

**FOSCE:** Formative Objective Structured Clinical Exam

**CAS**: Clinical Art and Sciences (Doctoring Course)

**PE:** Physical Exam

**FCC:** Foundations of Clinical Skills Course (Y1 & Y2)

**MeSH:** Medical Student Home (Y1 & Y2 Longitudinal Weekly Clinical Experience)

1. Demographic Data (For Research Only)

*Gender*

Male

Female

Other

2. Demographic Data (For Research Only)

*Age Range*

20-24 years old

25-28 years old

29-33 years old

34 years or older

3.Demographic Data (For Research Only)

*Highest Level of degree*

Bachelors

Masters

PhD

Other doctoral level degree

4.How many hours of training on eliciting a history had you experienced prior to enrolling at Netter?

None

0 to <2 hours

2 to < 4 hours

4 to <6 hours

> 6 hours

5.How many hours of training on conducting a physical examination had you experienced prior to enrolling at Netter?

None

0 to <2 hours

2 to <4 hours

4 to <6 hours

>6 hours

6.Identify your year of training

M1

M2

M3

M4

7. Indicate your level of agreement with the following

|  | Strongly Disagree | Disagree | Neutral | Agree | Strongly Agree |
| --- | --- | --- | --- | --- | --- |
| Implementation of an early identification program using FOSCE scores was a good decision by the CAS Course. |  |  |  |  |  |

8. Comment to above

9. Indicate your level of agreement with the following

|  | Strongly Disagree | Disagree | Neutral | Agree | Strongly Agree |
| --- | --- | --- | --- | --- | --- |
| Being identified was stigmatizing |  |  |  |  |  |

10. Comment to above

11. Indicate your level of agreement with the following

|  | Strongly Disagree | Disagree | Neutral | Agree | Strongly Agree |
| --- | --- | --- | --- | --- | --- |
| Being identified was beneficial |  |  |  |  |  |

12. Comment to above

13. Indicate your level of agreement with the following

|  | Strongly Disagree | Disagree | Neutral | Agree | Strongly Agree |
| --- | --- | --- | --- | --- | --- |
| Prior to being identified, I had concerns about my clinical skills performance |  |  |  |  |  |

14.Comment to above

15. Did being notified alter your approach in any way to OSCEs?

Yes

No

16. If Yes, did you independently (without formal coaching)

*Select all that apply*

Review your FOSCE videos

Work on your history taking

Work on your PE skills

Work on your communication skills

17.After being notified, did you attend a coaching session?

Yes

No

18.If Yes, who did you work with as your coach (outside of FCC class time)

*Select all that apply*

Your FCC Preceptor

Your MeSH Preceptor

Director of Clinical Skills Remediation

Director, Clinical Arts and Sciences Course

Assistant Director, Y2 Clinical Arts and Science Course

 Assistant Director, Y1 Clinical Arts and Science Course

19.Did you feel that coaching helped?

Yes

No

20.If yes, in which areas did you benefit?

*Select all that apply*

Clinical history taking

Physical exam

Patient Communication

Clinical Reasoning

Write-ups

Oral presentations

Confidence

Other

21.Did you talk to your MeSH preceptor about being identified?

Yes

No

22.Did you alter your approach to MeSH after being identified?

Yes

No

23.If Yes, how so?

24. If you did not contact someone to help coach you outside of class time, please tell us why.

*Select all that apply*

Didn't feel that I had the time

Didn't agree that I needed additional help

You practiced with a peer

Felt too embarrassed to ask for additional help

Thought I could correct any issues on my own

Other

25.How comfortable were you with your clinical skills prior to receiving any coaching outside of class time?

Select your answer

Very comfortable

Moderately comfortable

Slightly comfortable

Comfortable

Slightly uncomfortable

Moderately uncomfortable

Very uncomfortable

26. If you attended coaching, how comfortable were you with your clinical skills after receiving coaching outside of class time?

Select your answer

Very comfortable

Moderately comfortable

Slightly comfortable

Comfortable

Slightly uncomfortable

Moderately uncomfortable

Very uncomfortable

27.If you did not attend coaching, how comfortable were you with your clinical skills at the end of CAS Y2 Semester 2?

*For M2 students only*

Select your answer

Very comfortable

Moderately comfortable

Slightly comfortable

Comfortable

Slightly uncomfortable

Moderately uncomfortable

Very uncomfortable

28. For Future Consideration

*When a student is identified and sent an early notification email, who should be included in this email (check all that apply)?*

Course Director/Assistant Course Director

Director

Your FCC Preceptor

Dean of Student Affairs

Your MeSH Preceptor

Other

29. For Future Consideration

*When a student is identified, do you think that coaching should be*

Optional

Mandatory

30. For Future Consideration

*What changes, if any, would you make to the wording of the early identification email?*

31. Any other comments:
