# Supplementary material for: ‘Early identification of struggling pre-clerkship learners using formative clinical skills OSCEs: an assessment for learning program.’
Source: Med Educ Online. 2022 Jan 20;27(1):2028333. doi: 10.1080/10872981.2022.2028333 (PMC8786239; doi:10.1080/10872981.2022.2028333)
Supplement: Supplemental Material [file ZMEO_A_2028333_SM1695.zip › Supplementary files/Electronic Supplementary Material 2.docx]

Table 4: Linear Mixed Models Estimated Model Means (SEM)

| **Intervention** | **Before** | **After** | **P-Value** |
| --- | --- | --- | --- |
| Identified | 87.0 (0.4) | 89.0 (0.6) | .011 |
| Meeting |  |  | .480 |
| Participated in coaching | 86.4 (0.6) | 88.9 (0.7) |  |
| Did not participate in coaching | 87.5 (0.6) | 89.1 (0.8) |  |
