# Supplementary material for: ‘Early identification of struggling pre-clerkship learners using formative clinical skills OSCEs: an assessment for learning program.’
Source: Med Educ Online. 2022 Jan 20;27(1):2028333. doi: 10.1080/10872981.2022.2028333 (PMC8786239; doi:10.1080/10872981.2022.2028333)
Supplement: Supplemental Material [file ZMEO_A_2028333_SM1695.zip › Supplementary files/Electronic Supplementary Material 3.docx]

Table 5: Linear Mixed Models Parameter Estimates

| **Intervention** | **Coefficient** | **Std Error** | **P-Value** |
| --- | --- | --- | --- |
| Communication | 1.10 | 1.01 | .270 |
| History | 1.09 | 0.98 | .269 |
| Physical Exam | 4.47 | 1.21 | <.001 |
